# Transperitoneal vs. Retroperitoneal Approach in Laparoscopic Partial Nephrectomy for Posterior Renal Tumors: A Retrospective, Multi-Center, Comparative Study

**DOI:** 10.3390/jcm13030701

**Published:** 2024-01-25

**Authors:** Gonçalo Mendes, Mariana Madanelo, Fernando Vila, Rui Versos, Bernardo Lobão Teixeira, Maria Alexandra Rocha, Sofia Mesquita, Miguel Marques-Monteiro, Paulo Príncipe, Ricardo Ramires, Joaquim Lindoro, Avelino Fraga, Miguel Silva-Ramos

**Affiliations:** 1 Urology Department, Centro Hospitalar Universitário de Santo António, 4099-001 Porto, Portugal; goncalo.grilomendes@gmail.com (G.M.); marianacmadanelo@gmail.com (M.M.); bernardolat@gmail.com (B.L.T.); marialexandrarocha@gmail.com (M.A.R.); sofiaoplmesquita@gmail.com (S.M.); mmonteiro.iam@gmail.com (M.M.-M.); paulo.principe@gmail.com (P.P.); avfraga@gmail.com (A.F.); 2 Urology Department, Centro Hospitalar Tâmega e Sousa, 4564-007 Penafiel, Portugal; fernandovila1@gmail.com (F.V.); joaquim.lindoro@gmail.com (J.L.); 3 Urology Department, Hospital da Senhora da Oliveira—Guimarães, 4835-044 Guimarães, Portugal; rui.1971.versos@gmail.com (R.V.); ricardo.ramires@gmail.com (R.R.)

**Keywords:** laparoscopic partial nephrectomy, retroperitoneal, transperitoneal, laparoscopy, kidney cancer

## Abstract

**Purpose:** The aim of our study is to compare the perioperative, functional, and oncological outcomes of laparoscopic transperitoneal partial nephrectomy (LTPN) and laparoscopic retroperitoneal partial nephrectomy (LRPN) for posterior cT1 renal tumors. **Methods:** We retrospectively collected data on all patients who consecutively underwent LTPN and LRPN for posterior cT1 renal tumors in three different centers from January 2015 to January 2023. Patients with a single, unilateral, cT1 renal mass, located in the posterior renal surface were included. Patients’ data regarding perioperative, functional, and oncological outcomes were collected from medical records and statistically analyzed and compared. **Results:** A total of 128 patients was obtained, with 53 patients in the LPTN group and 75 patients in the LRPN group. Baseline characteristics were similar. Warm ischemia time (WIT) (18.8 vs. 22.6 min, *p* = 0.002) and immediate postoperative eGFR drop (−6.1 vs. −13.0 mL/min/1.73 m^2^, *p* = 0.047) were significantly lower in the LPTN group. Estimated blood loss (EBL) (100 vs. 150 mL, *p* = 0.043) was significantly lower in the LRPN group. All other perioperative and functional outcomes and complications were similar between the groups. The positive surgical margin (PSM) rate was lower in the LRPN group, although without statistical significance (7.2% vs. 13.5%, *p* = 0.258). Surgical success defined by Trifecta (WIT ≤ 25 min, no PSM, and no major postoperative complication) was similar between both approaches. **Conclusions:** LTPN has significantly shorter WIT and a significantly smaller drop in immediate eGFR when compared to LRPN for posterior renal tumors. On the other hand, LRPN has significantly less EBL than LTPN. LRPN demonstrated fewer PSMs than LTPN, albeit without statistical significance. In terms of overall surgical success, as defined by Trifecta, both approaches achieved similar results.

## 1. Introduction

Partial nephrectomy (PN) is a well-established surgical modality for cT1 renal masses (RMs) and is strongly recommended when technically feasible [1]. This procedure can be performed via the traditional open route, or alternatively, minimally invasive techniques might be considered, such as laparoscopic partial nephrectomy (LPN) or robot-assisted partial nephrectomy (RAPN) [2,3,4]. Considering that all these approaches have similar functional and oncologic outcomes [5,6,7], minimally invasive techniques are generally sought due to better perioperative outcomes, such as a decreased length of stay (LOS) and less estimated blood loss (EBL) [2,3,4,5,6,7,8]. Despite the rapidly growing adoption of RAPN, LPN remains a viable option, especially in centers not equipped with a robotic platform [9,10,11].

LPN can be performed by either a transperitoneal (TP) or retroperitoneal (RP) approaches, each of them with their advantages and disadvantages [12]. Laparoscopic transperitoneal partial nephrectomy (LTPN) provides better spatial orientation owing to more familiar landmarks and anatomical references and offers an increased working space with enhanced maneuverability. Also, port placement is easier due to a vast skin surface. However, it is challenging in posterior renal tumor dissection and reconstructive suturing and in patients with multiple previous abdominal surgeries. In contrast, laparoscopic retroperitoneal partial nephrectomy (LRPN) provides direct access to hilar structures and posterior RMs and obviates entering the peritoneum, benefiting patients with prior abdominal surgeries, and diminishing peritoneal contamination by blood or urine, therefore lowering the risk of peritoneal cavity irritation or peritonitis. However, it is difficult in obese patients with ample retroperitoneal fat, and in anterior RMs it poses ergonomic challenges for surgeons and requires a steep learning curve [13,14].

The aim of our study is to compare the perioperative, functional, and oncological outcomes of LTPN and LRPN for posterior cT1 RMs in three urological centers.

## 2. Materials and Methods

### 2.1. Patient Selection

We retrospectively collected data on all patients who consecutively underwent LTPN and LRPN for posterior cT1 RM in three different centers of the north of Portugal (Centro Hospitalar Universitário de Santo António, Centro Hospitalar Tâmega e Sousa and Hospital Senhora da Oliveira—Guimarães) from January 2015 to January 2023. All clinical and surgical data were collected from the patients’ medical records. The procedures were performed by three different teams of surgeons, one from each center. Each team consisted of a pair of surgeons, all of them with at least 5 years of staff experience. The choice of surgical technique was at the surgeon’s discretion. We included patients with a single, unilateral, cT1 renal mass, located in the posterior renal surface and for whom LPN was planned. We excluded patients with just anterior or neither anterior nor posterior RMs, cT2 or higher RMs, multiple tumors resected, metastatic disease, and use of the off-clamping technique. The study was approved by the Ethical Committees of each center.

### 2.2. Variables

Our database included information regarding age at surgery; gender; tumor laterality; body mass index (BMI); American Society of Anesthesiologists (ASA) score; pre- and postoperative serum hemoglobin (Hb) level; pre- and postoperative estimated glomerular filtration rate (eGFR), according to the Modification of Diet in Renal Disease (MDRD) formula [15]; tumor size; tumor complexity, evaluated by the R.E.N.A.L. Nephrometry score [16]; clinical stage; operative time (OT; minutes); warm ischemia time (WIT, minutes); estimated blood loss (EBL, mL); length of hospital stay (LOS, days); histology of surgical specimen; surgical margin status; a positive surgical margin (PSM), defined as the presence of tumor cells in the inked parenchymal margin; date of last appointment; recurrence; and postoperative complications, according to the Clavien–Dindo classification system [17]. These were further divided into minor (Clavien grade 1 and 2) and major complications (Clavien grade ≥ 3). Trifecta accomplishment, as a measure of surgical success, was defined as WIT ≤ 25 min, negative surgical margin, and no major postoperative complication.

### 2.3. Surgical Technique

For LRPN, the patient was placed in a full flank position, and access to the retroperitoneal cavity was gained through a small incision in the lumbar triangle with subsequent introduction of a Hasson trocar. Two additional ports (12 and 5 mm) were placed at the discretion of the surgeon, respecting triangulation principles. An additional 5 mm port for the assistant surgeon was also placed if necessary. Control of the renal artery or arteries was achieved with endobulldog clamps. The renal tumor was sharply excised with laparoscopic cold scissors. The closure of the entire tumor bed was performed with two layers of barbed suture. The renal artery was then unclamped and the bleeding of the tumor bed was carefully checked. Placement of collagen- or thrombin-based hemostatic agents at this phase was at the surgeon’s discretion. Lastly, an endobag was used to retrieve the specimen.

For LTPN, the patient was placed in a 30 degree elevated flank position. The pneumoperitoneum was established by insertion of a 12 mm camera trocar via a mini-laparotomy access. This port was placed peri-umbilical, or paramedian in obese patients. After, a 12 mm and a 5 mm working port were introduced at the discretion of the surgeon, in triangulation fashion. An additional 5 mm port for the assistant surgeon was also placed if necessary. For right-sided partial nephrectomies, a 5 mm port was introduced below the xyphoid for retraction of the liver. Dissection of the ascending and descending colon and the kidney was performed. Because only posterior tumors were evaluated, these often needed complete mobilization of the whole kidney. Renal artery or arteries were clamped by endobulldog clamps, and the renal tumor was sharply excised with laparoscopic cold scissors. Closure of the entire tumor bed was performed as in the LRPN approach, with two layers of barbed suture. The renal artery was then unclamped and bleeding of the tumor bed checked, with placement of hemostatic agents as for LRPN if necessary. The tumor was ultimately retrieved with an endobag. A perirenal drain was routinely left in place in all patients of both cohorts. There was no intraoperative frozen section analysis performed with either procedure. All procedures were performed in warm ischemia, given that no method of intracorporal cooling was used in any patient.

### 2.4. Statistical Analysis

Continuous variables with normal distribution are presented as mean ± standard deviation and compared by Student’s *t*-test, while continuous variables with non-normal distribution are presented as medians accompanied by interquartile ranges (IQRs) and compared by the Mann–Whitney-U test. Categorical variables are presented as proportions, and comparisons of two categorical variables are performed with the Pearson’s Chi-square test and Fisher exact test. Statistical analyses were conducted using SPSS Statistics version 27. All tests were two sided, and statistical significance was set at *p* < 0.05.

## 3. Results

A total of 128 patients was obtained, 53 patients in the LPTN group and 75 patients in the LRPN group. Baseline characteristics are summarized in Table 1. Age, BMI, ASA score, R.E.N.A.L score, tumor size, preoperative Hb, and preoperative eGFR were similar between both surgical approaches. Even though there were no differences between both groups, the LTPN group had a bigger proportion of cT1b tumors compared to the LRPN group, although without statistical significance.

Perioperative data are presented in Table 2. There was one conversion in the LTPN group, which was converted to radical nephrectomy due to hilar vessel injury, and six conversions in the LRPN group, all of them to open PN (*p* = 0.238), and these patients were excluded from further analyses. Operative time (127 min in LTPN group vs. 134 min in LRPN group, *p* = 0.347) and LOS (4 days in LTPN group vs. 4 days in LRPN group, *p* = 0.619) were similar between both approaches, and also postoperative Hb and variation in pre- and postoperative Hb were similar between groups. WIT was significantly shorter in the LTPN group (18.8 vs. 22.6 min, *p* = 0.002), and variation in pre- and postoperative eGFR at discharge was significantly less in the LTPN group (−6.1 vs. −13.0 mL/min/1.73 m^2^, *p* = 0.047); however, eGFR at 6 months postoperatively was similar between the groups (−3.3 mL/min/1.73 m^2^ in the LTPN group vs. −7.8 mL/min/1.73 m^2^ in the LRPN group, *p* = 0.109). EBL was significantly less in the LRPN group (100 vs. 150 mL, *p* = 0.043). The overall complication rate was similar between the groups (15.4% in LTPN vs. 15.9% in LRPN group, *p* = 0.934), as were minor (7.7% in LTPN group vs. 11.6% in LRPN group, *p* = 0.552) and major complications (7.7% in LTPN group vs. 4.3% in LRPN group, *p* = 0.461). The most common minor complication was postoperative fever which was managed conservatively with antipyretics or antibiotics. Regarding major complications, in the LTPN group, there were three pseudoaneurysms with need for selective angioembolization, and one patient had a large peri-renal hematoma with the need for percutaneous drainage; in the LRPN group, there were two pseudoaneurysms with need for selective angioembolization and one patient with ureteral obstruction by clots with the need for placement of a ureteral stent.

The oncological data and overall success rates are summarized in Table 3. The mean follow-up was 37.4 months in the LTPN group and 33.6 months in the LRPN group (*p* = 0.388). The proportion of benign lesions was similar between groups (21.2% in the LTPN group vs. 27.5% in the LRPN group, *p* = 0.421). Despite there being no statistically significant difference, the PSM rate was higher in the LTPN group (13.5% vs. 7.2%, *p* = 0.258). Overall success, measured by Trifecta, was similar between groups (71.2% in the LTPN group vs. 65.2% in the LRPN group, *p* = 0.489). The recurrence rate was similar between groups (2.4% in LTPN group vs. 4.0% in LRPN group, *p* = 1.000). There was one (2.4%) recurrence in the LTPN group, in a patient with a clear-cell renal carcinoma, stage pT1a, with a grossly invaded margin who had a local recurrence within less than one year of follow-up, and who subsequently underwent radiofrequency ablation; there were two (4.0%) recurrences in the LRPN group, both in patients with a clear-cell renal carcinoma, stage pT1a, with negative margins, one with a local recurrence within one year from the first surgery who subsequently underwent radical nephrectomy, and the other with distant metastasis, who subsequently was started on systemic therapy.

## 4. Discussion

Open PN was considered for a long time the gold standard for surgically managing localized renal masses suitable for conservative surgery, and it remains a viable option in challenging cases [1]. However, over the last few decades, minimally invasive approaches to PN have gained recognition as safe, feasible, and effective alternatives to open surgery. These approaches offer several established advantages, including reduced postoperative pain, resulting in shorter hospital stays and lower complication rates. This paradigm shift underscores the evolving landscape of renal mass surgery, where minimally invasive techniques now stand as viable and advantageous options alongside the traditional gold standard of open PN [2,3,4,5]. Still, the best surgical approach of LPN for posterior tumors of the kidney remains a topic of debate. Despite a lot of comparative studies between both approaches in the entirety of renal tumors [12,18,19,20,21], only a few papers compare both techniques for exclusively posterior tumors [13,22]. To the best of our knowledge, this is the largest multicenter comparison of TP and RP approaches on pure LPN for posterior renal tumors. In the present study, we demonstrated a shorter WIT and a better immediate postoperative renal function preservation in the LTPN group, whereas LRPN demonstrated less EBL. Postoperative renal function at 6 months postoperatively was similar between both approaches. All the other outcomes were similar between the groups, despite fewer PSMs in the LRPN group.

Most of the previous studies comparing both approaches for all tumor locations show advantages of the LRPN [12,18,19,20,21], either when anterior and posterior tumors are similarly distributed between techniques [12,18] or when anterior tumors are primarily approached by LTPN and posterior tumors by LRPN [19,23]. Meta-analysis has demonstrated shorter OT, lower EBL, and shorter LOS in favor of LRPN [20,21]. Studies comparing both approaches solely for posterior tumors demonstrated shorter OT [13,22], shorter WIT, and less EBL [23] with the RP approach. Our study demonstrated comparable OT and LOS for both approaches, but we did demonstrate less EBL for the LRPN group. Comparable LOS might reflect similar perioperative care and discharge criteria, even among different hospitals [13]. Similar OT might reflect the fact that matched analysis was not performed, and a selection bias is a possibility [24]. A possible explanation for less EBL in the LRPN group might be because of the overall less need for mobilization of other structures, such as the colon, and less extensive dissection of the renal tumor, as the approach is posterior and the tumor is encountered more easily, unlike in the LTPN approach in which mobilization of the whole kidney is often needed to expose a posterior tumor. Regarding complications, both techniques had comparable rates of overall, minor, and major complications, which is in line with previous studies [13,21,22]. Li et al. [22], in a comparison of LPN by either the RP or TP approach, and for either posterior and anterior tumors, reported a rate of major complications (Clavien Dindo grades ≥ 3) in the subset of patients with purely posterior tumors of 3.7% for the LRPN approach, which is similar to the value of 4.3% obtained in our study, and 4.7% for the LTPN approach, a value slightly lower than the 7.7% demonstrated in our study. In any case, the major complication rate of LTPN for posterior renal tumors in this study was well below that achieved by other authors [13], and more importantly, it was similar for both approaches.

WIT is one of the biggest concerns of surgeons when performing PN. Initially thought to be of major importance for renal function preservation [25], the role of WIT as the most important factor in the deterioration of renal function has been questioned [26]. Other factors, such as parenchymal mass preservation, might be important, as recovery of renal function is strongly and proportionately correlated to the parenchymal mass saved [27]; still, efforts to limit WIT have translated to better preservation of renal function after PN, which reflects the importance of limited WIT in achieving better functional results [26]. In our study, LTPN demonstrated a significantly shorter WIT and a significantly smaller drop in immediate postoperative eGFR after surgery, translating to better immediate preservation of renal function; these findings highlight the importance of shorter WIT for renal preservation. Still, when comparing eGFR at 6 months postoperatively, even though LTPN has a smaller drop than LRPN, this difference is not statistically significant. One might hypothesize that the immediate drop in renal function could be due to the immediate insult of the surgical procedure and that warm ischemia reflects more on renal function in the short term, but that in the medium term, this effect is less important. Nevertheless, it is important to mention that there was no matched analysis in our study, which might have biased the results. Also, comorbidities between both groups were not accounted for, and this is also an important contributor to renal preservation after PN [28]. In fact, Flammia et al. demonstrated that patients with hypertension undergoing minimally invasive PN for cT1 renal tumors had worse renal function outcomes when compared to their counterparts without hypertension. Moreover, other comorbidities such as diabetes could further impair renal function [29]. In our study, we could not identify previous comorbidities for the majority of patients due to incomplete registry of data, and this represents a limitation to our study.

PSM rate is another concern when performing LPN, mainly related to oncological outcome. While PSMs may increase the risk of recurrence, their specific impact on the progression of disease is still a subject of ongoing research. Other factors, such as a tumor’s stage, grade and multifocality, could be more pivotal factors than PSMs in determining the likelihood of local or distal recurrence [30,31]. Even though the PSM rate was not significantly different between groups, it is worth noting that the LRPN group had fewer overall PSMs. One may hypothesize that dissection of posterior tumors might be facilitated through the RP approach, while in the TP approach, the mobilization and rotation of the whole kidney may render the dissection and removal of the tumor more difficult, causing a higher likelihood of positive margins. Nevertheless, the recurrence rate was similar between the groups, and in fact, it is interesting to note that two out of the three recurrences observed in the whole sample where in patients without PSM on final pathology, which further highlights the importance of other factors for recurrence [30,31]. When accounting for surgical quality, based on Trifecta as a surrogate marker (defined in this study as WIT ≤ 25 min, no PSM, and no major postoperative complication), both groups had similar outcomes. Although Trifecta and Pentafecta were compared between both approaches previously [32], they had not been compared in previous studies evaluating only posterior tumors [13,22,33,34,35,36]. In our study, Trifecta was similar between groups, highlighting the overall comparable outcomes between both surgical approaches, despite the differences found in WIT and PSM rate.

There is some published literature on RAPN, namely on comparisons between the RP and TP approaches for posterior renal tumors [33,34,35,36]. Kim et al. [33] demonstrated a shorter LOS for patients with posterior renal tumors approached with the RP approach when compared with the TP approach. These findings were corroborated by Paulucci et al. [34], and their study also demonstrated a shorter operative time in the RP-RAPN group. Maurice et al. [35] also demonstrated a shorter LOS for the RP approach, but on the other hand, found that the RP approach was associated with a longer WIT, as was the case in our study. Nevertheless, all other outcomes were similar between RP-RAPN and TP-RAPN. More recently, a systematic review and meta-analysis conducted by McLean et al. [36], which included the three previously mentioned studies, demonstrated that RP-RAPN had a shorter LOS than TP-RAPN, with all other outcomes of interest, such as WIT, OT, EBL, and PSM rate, being equal between groups. These findings are in divergence with those in our study, in which we demonstrated a shorter WIT and smaller drop in eGFR in the TP approach and, on the other hand, a smaller EBL in the RP approach. As previously stated, none of these studies focused on surrogates of surgical quality, like Trifecta, which in our study, was similar between both surgical approaches.

Our study has several limitations that warrant acknowledgment. The main shortcomings are those inherent to the retrospective design. Patients were included from three different centers and at different times; therefore, there was no standardization for the choice of surgical technique. Also, there were three different teams of surgeons, one from each center, performing the surgeries, and in one of the centers, only the RP approach was used, while in the other two centers, both approaches were used, at the surgeons’ discretion; this might have influenced the results, despite all teams being composed of experienced surgeons. Another important caveat, as previously stated, is the fact that comorbidities were not accounted for, as this could have had an impact on the perioperative outcomes and renal function outcomes. Finally, there was no matched pair analysis in our study, and so, even though the baseline characteristics were overall comparable, heterogeneity between the groups is a possibility, which may have biased the results.

## 5. Conclusions

Our study demonstrated that LTPN has significantly shorter WIT and a significantly smaller drop in immediate eGFR when compared to LRPN for posterior renal tumors. The eGFR at 6 months postoperatively, however, was similar between both surgical approaches. On the other hand, LRPN has significantly less EBL than LTPN. Despite not achieving statistical significance, it is also worth mentioning that LRPN demonstrated fewer PSMs than LTPN. In terms of overall surgical success, as defined by Trifecta, both approaches achieved similar results. In summary, both approaches are valid for posterior renal tumors in experienced hands. Further studies, mainly prospective comparative studies, are needed in the future to add more information on which approach is best suited for small posterior renal tumors.

## Figures and Tables

**Table 1:** Baseline characteristics of the groups.

	LTPN (*n* = 53)	LRPN (*n* = 75)	*p*
Age (years)	63.0 ± 11.0	60.6 ± 11.5	0.232
BMI (kg/m^2^)	27.9 ± 4.0	27.3 ± 4.0	0.408
ASA score	2.0 (2.0–3.0)	2.0 (2.0–3.0)	0.845
Female sex (*n*, %)	23 (43.4%)	21 (28.0%)	0.071
Right-sided tumor (*n*, %)	27 (50.9%)	39 (52.0%)	0.906
R.E.N.A.L score	6.0 (5.0–7.5)	6.0 (5.0–8.0)	0.720
Tumor size (mm)	30.9 ± 10.9	28.9 ± 8.7	0.240
cT1b (*n*, %)	11 (20.8%)	7 (9.3%)	0.067
Preop. Hb (g/dL)	14.0 ± 1.5	13.9 ± 1.4	0.964
Preop. eGFR (mL/min/1.73 m^2^)	80.1 ± 23.6	81.6 ± 20.4	0.711

ASA—American Society of Anesthesiologists; BMI—body mass index; eGFR—estimated glomerular filtration rate; Hb—hemoglobin; LRPN—laparoscopic retroperitoneal partial nephrectomy; LTPN—laparoscopic transperitoneal partial nephrectomy. Values with normal distribution are presented as mean ± standard deviation, while values with non-normal distribution are presented as median (interquartile range). Categorical variables are presented as proportions.

**Table 2:** Perioperative data of the groups.

	LTPN (*n* = 52)	LRPN (*n* = 69)	*p*
Conversion (*n*, %)	1/53 (1.9%)	6/75 (8.0%)	0.238
Postop. Hb (g/dL)	12.2 ± 1.5	12.1 ± 1.3	0.895
Variation of Hb pre- and postop. (g/dL)	1.8 ± 1.0	1.9 ± 1.1	0.615
Variation of eGFR preop. and at discharge (mL/min/1.73 m^2^)	−6.1 ± 20.3	−13.0 ± 17.5	0.047
Variation of eGFR preop. and at 6 months (mL/min/1.73 m^2^)	−3.3 ± 13.4	−7.8 ± 15.0	0.109
Operative time (min)	127 ± 32	134 ± 47	0.347
Estimated blood loss (mL)	150.0 (50.0–300.0)	100.0 (50.0–225.0)	0.043
WIT (min)	18.8 ± 5.7	22.6 ± 6.8	0.002
Length of stay (days)	4.0 (3.0–6.0)	4.0 (3.0–6.0)	0.619
Overall complications (*n*, %)	8 (15.4%)	11 (15.9%)	0.934
Minor complications, grades 1 & 2 (*n*, %)	4 (7.7%)	8 (11.6%)	0.552
Major complications, grade ≥ 3 (*n*, %)	4 (7.7%)	3 (4.3%)	0.461
Transfusion (*n*, %)	0 (0%)	2 (2.9%)	0.506

eGFR—estimated glomerular filtration rate; Hb—hemoglobin; LRPN—laparoscopic retroperitoneal partial nephrectomy; LTPN—laparoscopic transperitoneal partial nephrectomy; WIT—warm ischemia time. Values with normal distribution are presented as mean ± standard deviation, while values with non-normal distribution are presented as median (interquartile range). Categorical variables are presented as proportions.

**Table 3:** Oncological data and overall surgical success of the groups.

	LTPN (*n* = 52)	LRPN (*n* = 69)	*p*
Follow-up (months)	37.4 ± 22.8	33.6 ± 17.5	0.388
Benign lesions (*n*, %)	11 (21.2%)	19 (27.5%)	0.421
PSM (*n*, %)	7 (13.5%)	5 (7.2%)	0.258
Trifecta (*n*, %)	37 (71.2%)	45 (65.2%)	0.489
Recurrence (*n*/total, %)	1/41 (2.4%)	2/50 (4.0%)	1.000

LRPN—laparoscopic retroperitoneal partial nephrectomy; LTPN—laparoscopic transperitoneal partial nephrectomy; PSM—positive surgical margin. Values with normal distribution are presented as mean ± standard deviation, while values with non-normal distribution are presented as median (interquartile range). Categorical variables are presented as proportions.

## Data Availability

No new data were created or analyzed in this study. Data sharing is not applicable to this article.
